# Dairy Victory Platform: A novel benchmarking platform to empower economic decisions on dairy farms

**DOI:** 10.3168/jdsc.2024-0617

**Published:** 2024-10-11

**Authors:** E.N.A. Freitas, V.E. Cabrera

**Affiliations:** 1 Informatic Department, Federal Institute of Goiás, Goiania, Brazil, 76600000; 2 Animal and Dairy Sciences Department, University of Wisconsin–Madison, Madison, WI 53706

## Abstract

In the face of diminishing economic margins, dairy farmers globally are compelled to maintain economic competitiveness. Benchmarking emerges as a strategic tool to establish new, achievable improvement objectives that balance ambition with practicality. This typically requires integrating diverse data sources, such as feed, milk production, diet, and market prices. However, many farms lack essential cow-level data like daily milk output and milk income over feed cost (IOFC), which hampers economic performance visibility and informed decision making. To address this challenge, we introduce Dairy Victory Platform (DVP), a novel cloud-based benchmarking platform designed to simplify complex analyses. The DVP uniquely calculates zootechnical and economic key performance indicators (KPI) at the cow and herd levels and benchmarks these against a dynamically selected cohort of farms, facilitating comparisons across various farm sizes, and milk production levels. The primary objective of this paper is to demonstrate the utility of DVP as a decision-making tool in supporting farmers and consultants with both operational and strategic decisions. It emphasizes leveraging benchmarking information to enhance decision-making processes, thereby highlighting the significant value that DVP brings to farmers, consultants, and dairy farm stakeholders. Our study analyzed data from 712 farms from December 2023, focusing on several KPI, such as milk production, milk quality, feed efficiency, and IOFC using DHI records. This approach showcases DVP's ability to use minimal data inputs for detailed analysis, leveraging peer performance to set desirable and achievable goals. We present a case study demonstrating how the DVP platform can guide dairy farms using anonymized peer data. This approach enables users to potentially improve their IOFC by up to 35%. Our findings highlight the potential of DVP as a powerful tool for generating insightful analyses, simulations, and recommendations, primarily from test-day data, but also integrated with market and estimated data. This supports more strategic decision-making in dairy management, including automatic goal-setting based on peer performance.

## Highlights

• DVP benchmarks KPI such as FE, IOFC, and margin, with no user input needed.
• DVP helps farms set goals based on peer or self-performance, boosting FE and IOFC.

## Graphical Abstract

**Figure:**
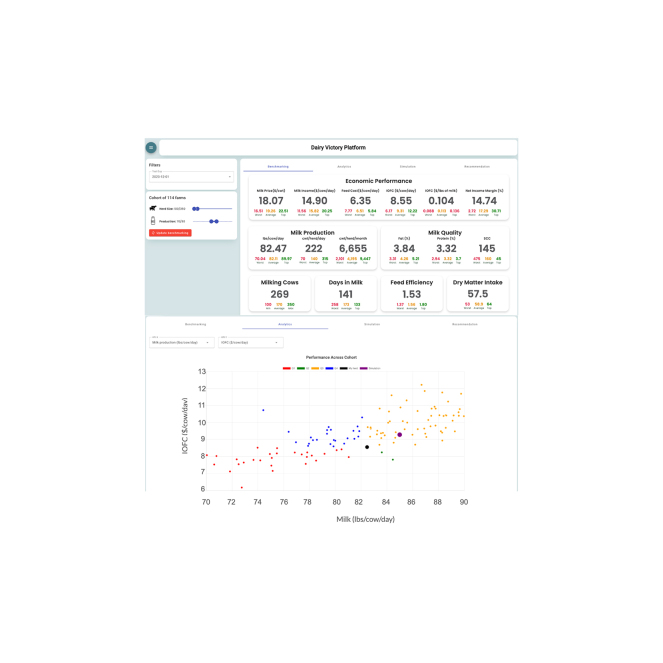
**Summary:** The Dairy Victory Platform (DVP) addresses the critical gap in accessible, qualified economic information for dairy farms. It leverages monthly test-day data from Dairy Herd Improvement-processing centers and commodity market prices. By considering the geographic locations of farms, the DVP calculates vital decisionmaking key performance indicators (KPI) such as feed efficiency (FE), milk income over feed cost (IOFC), and net income margin. This enables zootechnical and economic benchmarking of farms. Designed for dairy farmers seeking to automate performance measurement and analysis, the DVP offers potential insights for economic enhancement, moving beyond stressful and often imprecise manual data analysis. The platform's architecture is robust and sophisticated, encompassing data integration, benchmarking, analytics, simulation, and recommendation modules.

In recent years, dairy farms have increasingly embraced technology, which has substantially enhanced data availability. Despite these advancements, effective benchmarking of dairy farm performance continues to pose significant challenges. These challenges primarily arise from 3 key issues: the integration of data from diverse sources is frequently inadequate; standardized metrics for comparison are not consistently applied across the industry; and there is a general scarcity of comprehensive economic performance data and achievable measures for assessing economic outcomes.

Several studies have provided insightful information from dairy data. Ramsbottom et al. (2021) found a strong positive correlation between the frequency of financial benchmarking and improvements in both technical and financial efficiency. In another study, Wolf et al. (2020) examined how profitability varies over time and across different herd sizes among dairy farms in the Upper Midwest United States. Despite these insights, there remains a critical need for a systematic and dynamic approach to effectively evaluate a diverse range of farms. This method should benefit farmers, consultants, DHI, and other stakeholders by identifying management weaknesses relative to peers that can be improved.

Benchmarking is an essential tool for understanding the operational performance of dairy farms and is pivotal in developing growth strategies (Hansen et al., 2015). This method provides detailed insights into each farm's strengths and areas needing improvement, helping to pinpoint both opportunities and challenges. Through careful analysis of key performance metrics, such as milk production and quality (fat, protein, SCC), feed efficiency (**FE**), and milk income over feed cost (**IOFC**), farms can make informed decisions. These decisions affect strategic areas including culling practices, nutritional planning, genetic enhancements, and expansion initiatives.

A comprehensive analysis of peer performance via benchmarking enables the establishment of realistic goals and the creation of actionable, well-structured development plans designed to promote sustainable growth. Additionally, benchmarking aids in prioritizing initiatives, allocating resources efficiently, and monitoring progress effectively. As a result, it can substantially improve decision-making capabilities and strengthen long-term market success.

The Dairy Victory Platform (**DVP**) has been developed to harness the power of benchmarking for dairy farm decision-making. This paper introduces the DVP platform and its components, culminating in a case study that illustrates how DVP can guide dairy farmers toward both progressive and achievable goals by benchmarking farm performance. Although DVP can empower economic decisions on dairy farms, it is important to hold its users responsible for the correct and proper management analysis of dairy farming operations and for avoiding inappropriate, nonsensical, and common mistakes, as emphasized by Ferris and Malcolm (1999). The DVP offers a solution for farmers who seek to automate the measurement and analysis of their performance, providing reliable economic insights and avoiding the pitfalls of stressful and inaccurate manual data processes. To achieve this, the DVP incorporates a sophisticated and robust computational architecture with 5 key modules: Data Integration (**DVP-DI**), Benchmarking (**DVP-B**), Analytics (**DVP-A**), Simulation (**DVP-S**), and Recommendation (**DVP-R**).

Dairy Victory Data Integration (DVP-DI): With dairy farm data volumes growing exponentially, integrating diverse data streams can be complex, tedious, and error-prone. Designed with big data principles, DVP-DI efficiently processes, stores, and analyzes large datasets. Drawing on our experience from projects like Dairy Brain (Cabrera et al., 2020), DVP-DI ingests test-day files from a large number of herds, using widely recognized data formats from the Council on Dairy Cattle Breeding (**CDCB**; https://uscdcb.com), provided by DHI associations. To facilitate economic analysis, DVP dynamically collects and integrates spot prices for various commodities from multiple markets.

Dairy Victory Benchmarking (DVP-B): DVP ensures all data remains anonymized, presenting key performance indicators (**KPI**) in a strategic framework as illustrated in the graphical abstract of this paper. We have developed a component within DVP that computes and stores a suite of KPI for each farm, using data processed by DVP-DI. This allows for sophisticated analysis aligned with strategic objectives. A modern, responsive, web-based dashboard enables farmers to visually discern associations among KPI.

Comparisons among farms are challenged due to the unique contexts and challenges each farm faces. To address this, the DVP platform enables users to benchmark farms against a configured cohort, which the user has 2 options to define. In the first option, the user is free to define the farms participating in the cohort by selecting the farms based on any combination of both number of cows and the average milk production of the herds, as shown in the graphical abstract. As an alternative option, the user can choose a predefined cohort, to which the evaluated farm has similarities based on some features as shown in Table 1. To provide these predefined more consistent cohorts, the DVP clusters farms based on their similarities across important KPI using a constrained K-means clustering algorithm, ensuring an equal number of farms in each cohort. All farm data are anonymized and no additional data input is required for streamlining the benchmarking process.

**Table 1:** Classification of farms into cohorts based on key performance indicators in December 2023^1^

Cohort	Farms (n)	Cows/farm (n)	Lactation no.	Age (d)	Milk (lb/cow per d)	Fat (%)	Protein (%)	SCC (×1,000)	DMI (lbs/cow per d)	FE	Milk price ($/cwt)	Feed cost ($/cow per d)	IOFC ($/cow per d)
1	89	78	2.35	1,519	71.7	4.32	3.37	305	54.7	1.47	19.26	6.02	7.71
2	89	76	2.59	1,661	75.8	4.13	3.28	135	56.0	1.48	18.89	6.17	8.10
3	89	77	3.47	2,034	76.2	4.29	3.33	202	56.4	1.49	19.31	6.23	8.42
4	89	76	2.36	1,498	80.1	4.22	3.29	112	57.8	1.53	19.21	6.38	8.94
5	89	108	2.04	1,320	82.2	4.27	3.31	111	58.7	1.56	19.38	6.49	9.41
6	89	271	2.49	1,509	86.5	4.32	3.34	145	60.0	1.61	19.52	6.63	10.20
7	89	1,781	2.37	1,404	88.9	4.36	3.37	155	61.4	1.63	19.66	6.93	10.50
8	89	536	2.39	1,427	91.6	4.31	3.30	120	61.9	1.66	19.47	6.87	10.88

1 Units are given intentionally as normally used by farmers (1 lb = 0.454 kg; 1 cwt = 45.4 kg). IOFC = milk income minus feed cost.

The user interface displayed in the graphical abstract (top panel) facilitates easy comparisons within these cohorts, highlighting the user's farm performance in bold black, and displaying the worst, average, and best performances from the selected cohort. The DVP automatically computes a set of crucial KPI at the cow level (including milk production, fat, protein, SCC, feed cost, FE and IOFC) and then aggregates these at the farm level. This consolidation allows us to benchmark farms and ensure consistency, quality, and compatibility on comparisons across both time and farms, which are vital in a big data environment. Because test-day reports do not include diet composition and associated costs, we approximate feed costs using market economic information and the physiologic performance of the cows for a reasonable economic benchmarking analysis. This process required an interactive and continuous validation with a pilot group of farms to balance both data simplicity, precision, and accuracy. These farms provided KPI that are well known to most farmers, such as DMI, feed cost, and IOFC. We specifically focused on validating feed cost and milk price, which are critical for deriving accurate KPI like IOFC. Additionally, a large DHI association that processes thousands of farms' records monthly reviewed and validated the historical KPI from a diverse group of farms, ensuring that our estimates were reasonable and applicable across various operations. In addition, as a first step to allow benchmarking and ensure consistent comparisons given the variety of premium schedules for milk pricing, we used class III milk prices as a baseline for all milk compensation, together with farm-specific fat, protein, and SCC performance. This standardization extends to SCC compensation and premiums for milk volume, fat, and protein. Feed efficiency is calculated based on the ratio of ECM to DMI, assuming 3.5% fat and 3.2% protein.

Dairy Victory Analytics (DVP-A): DVP-A enables visualization of farm performance across any 2 KPI through a dynamic, the input-output analysis principle from data envelopment analysis (DEA; Cooper et al., 2011), as illustrated in Figure 1. Farms are categorized into 4 quadrants to compare their performance of the current farm (marked in black) against: farms performing worse in both KPI (marked in red), farms performing worse in the x-axis KPI but better in the y-axis KPI (marked in blue), farms performing better in the x-axis KPI but worse in the y-axis KPI (marked in green), and farms performing better in both KPI (marked in yellow). The application of DEA principles facilitates insightful analysis, allowing for an effective comparison across any chosen pair of KPI supporting simulations and sensitivity analysis of potentially achievable performances (e.g., purple dot in graphical abstract).

**Figure 1:**
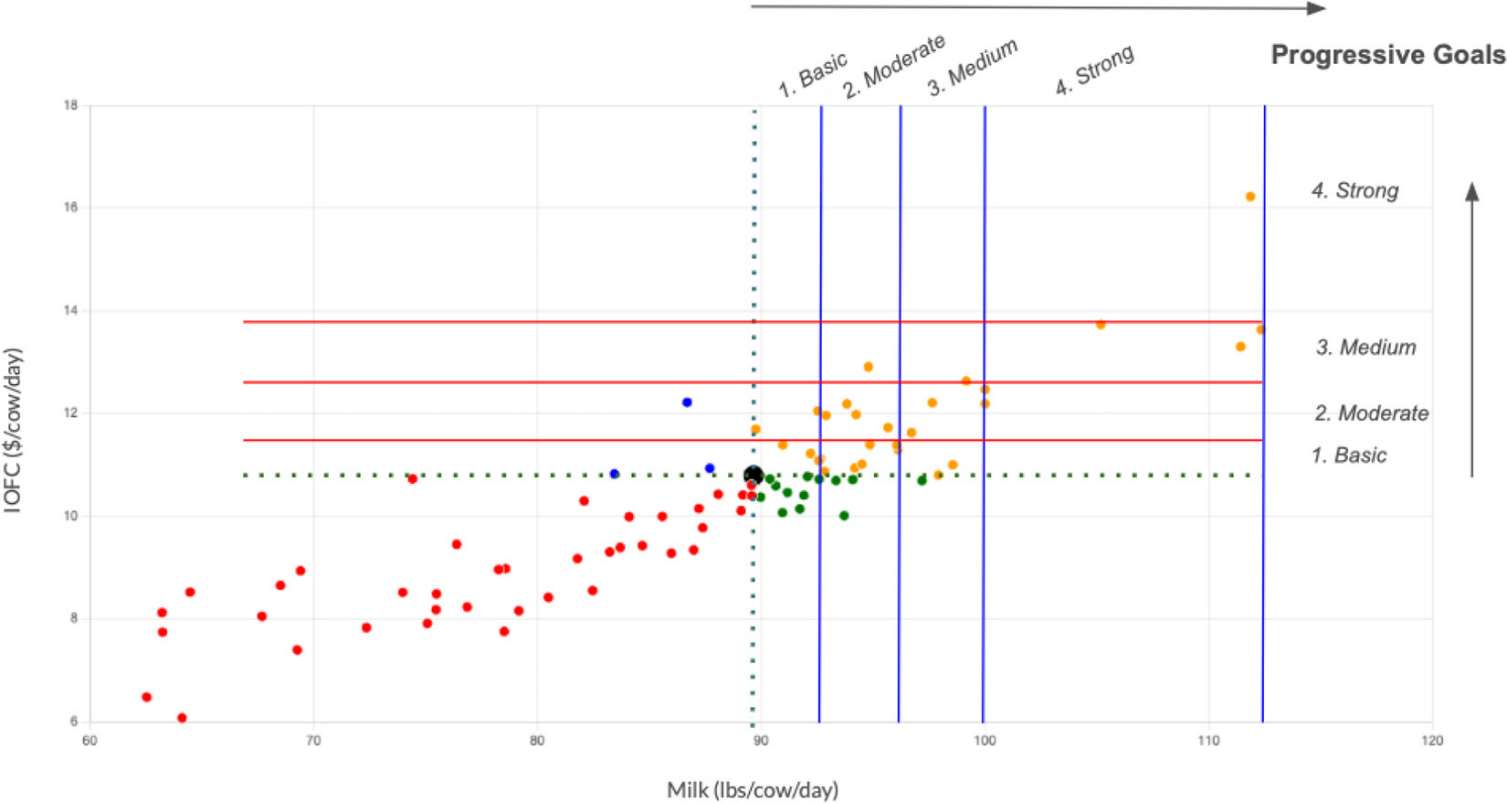
Correlation of milk production (x-axis) and milk income over feed cost (IOFC) across 85 farms, as analyzed by the Dairy Victory Platform. Red lines mean the conceptual limits of the progressive goals related to IOFC (y-axis), and blue lines mean the limits of the progressive goals regarding milk production (x-axis). Each data point indicates a separate farm. The farm under evaluation is marked with a large black data point. The color coding for other farms indicates their performance relative to the evaluated farm. Yellow-colored farms, termed “candidate farms,” serve as benchmarks for setting progressive goals for the evaluated farm at basic, moderate, medium, and strong levels. Farms performing worse in both KPI are indicated in red, farms performing worse in the x-axis KPI and better in the y-axis KPI are indicated in blue, and farms performing better in the x-axis KPI but worse in the y-axis KPI are marked in green. We found that farms in the strong levels remained consistently in the top 25% across all months of the 2023 database.

Dairy Victory Simulation (DVP-S): the DVP research team, along with pilot farms we enrolled to evaluate our tool, recognized the need to assess the economic impact of strategic changes. Dairy Victory Simulation offers the ability to simulate adjustments in key variables under farm control and in reference with the cohort farms. These variables include milk production, fat and protein content, SCC, and diet cost. Using insights from the benchmarking and analytics modules, farms can explore the effects of these changes on the entire herd or on specific lactation groups, providing a clear view of potential outcomes from proposed modifications.

Dairy Victory Recommendations (DVP-R): In addition to the capacity of integrating data and benchmarking farms, the DVP is also able to suggest progressive goals automatically. This mechanism uses real benchmarking data from similar farms to outline a feasible and logical path for growth. This approach ensures that the proposed goals are achievable, as they are already being met by other farms within the same cohort. Guided by data-driven algorithms, the DVP explores complex relationships within a multidimensional database to provide insights and practical recommendations, supporting farm improvements based on the performance of similar peers.

The DVP-R automatically suggests progressive and achievable goals for economic improvements based on the performance of candidate farms within their cohort. Candidate farms are those performing better than the farm under evaluation but not necessarily the best in the cohort. Based on the performance of these candidate farms, the DVP-R establishes 4 goal levels: basic (first quartile), moderate (mean), medium (third quartile), and strong (maximum values), as illustrated in Figure 1. Recommendations and suggestions from cohort groups further enrich the DVP-R, allowing users to apply tailored strategies for improvement.

Additionally, the DVP-R calculates the average economic impact of reaching these goals. We focused on milk production, fat, protein, SCC, and feed cost as critical KPI that directly affect dairy farm economics and are under farmer management control. For instance, data from December 2023 indicates that milk yield improvement goals for an evaluated farm (Figure 1) can range from a 5.8% increase at the basic level to a 47.3% increase at the strong level, with corresponding economic impacts ranging from 8.0% to 65.3%. Similarly, feed cost reduction goals suggest possible savings from 3.5% to 30.8%, with IOFC impacts ranging from 2.4% to 30.8%. Given the complexity of farm management, it is prudent for these goals to be set in a gradual manner, starting from basic and progressively advancing to higher levels. Although these proposed goals provide a valuable starting point, farmers should also consider other critical aspects of their operations (such as financial, genetic, nutritional, and management factors) before committing to specific targets.

To demonstrate the potential of the DVP platform, we conducted a case study that used the DVP modules to assist dairy farmers in setting strategic goals through benchmarking against similar farms. For this purpose, we used data from AgSource DHI milk recording (https://agsource.com), including test-day reports from December 2023. This data generated a dataset of cow-level KPI for cows undergoing CDCB genetic evaluation. For this analysis, we included only farms with at least 30 milking cows. The final dataset comprised 712 farms and 265,486 milking cows monthly by the end of the study period, which were benchmarked against cohort farms selected by the constrained K-means clustering algorithm (Table 1).

The economic impact by reaching the suggestions can vary depending on the level of goals assumed. To exemplify the potential of the platform, if a farm with a current fat percentage of 3.9% reaches the “moderate” goal of 4.3% (an increase of 11.3%) this would result in a 7.6% rise in IOFC, escalating from $11.06 to $12.30 per cow per day. The economic advantages of strategic performance improvements are showcased in Table 2, which elaborates on the enhancements in IOFC for all proposed progressive goals. Table 2 is central to understanding these dynamics of the progressive goals recommendation. It compares the current performance of a farm with the performance benchmarks of candidate farms in its cohort, detailing the potential growth and economic impact of achieving each level of progressive goals. The farm's actual FE in this example is 1.53, and the progressive suggested targets range from 1.56 (basic) to 1.80 (strong). The current IOFC is $11.06, and the basic, moderate, medium, and strong IOFC goals according to the cohort farms are $11.44, $13.16, $13.87, and $20.17, respectively.

**Table 2:** Comparison of a farm's key performance indicators (KPI) against cohort benchmark levels (basic, moderate, medium, and strong), and economic impact upon achieving the recommended progressive goals^1^

KPI	Farm	Cohort performance				IOFC impact (%)			
		Basic	Moderate	Medium	Strong	Basic	Moderate	Medium	Strong
Milk (lb/cow per d)	85.6	89.4	94.9	97.6	113.6	4.3	14.4	19.2	44.2
Fat (%)	3.89	4.06	4.32	4.54	5.56	3.06	7.65	10.61	38.42
Protein (%)	3.18	3.28	3.37	3.41	3.90	1.72	3.58	4.40	14.16
SCC	233	159	129	92	60	1.18	11	2.25	3.75
Feed cost ($/cwt)	7.20	7.04	6.59	6.28	5.32	1.42	51	8.28	16.98
Feed efficiency	1.53	1.56	1.60	1.64	1.80	—	—	—	—
IOFC ($/cow per d)	11.06	11.44	13.16	13.87	20.17	—	—	—	—

1 Units are given intentionally as normally used by farmers (1 lb = 0.454 kg; 1 cwt = 45.4 kg). IOFC = milk income minus feed cost.

There are several benefits to dairy farmers of using platforms like the DVP. In addition to its ability to generate and compare important KPI for decision making and recommendations based on peer performance effectiveness, DVP provides an easy and user-friendly way to simulate and analyze the impact of hypothetical change scenarios. The usability and benefits of the DVP have been validated regarding diet costs and milk prices using AgSource dataset, but it is currently available for use with any DHI dataset or similar datasets that want to explore the zootechnical and economic benefits of dairy benchmarking using test-day data.
